# Spatially variable habitat quality contributes to within-population variation in reproductive success

**DOI:** 10.1002/ece3.1427

**Published:** 2015-03-06

**Authors:** Blaine D Griffen, Alexandra P Norelli

**Affiliations:** 1 Department of Biological Sciences, University of South CarolinaColumbia, South Carolina, 29208; 2 Marine Science Program, University of South CarolinaColumbia, South Carolina, 29208

**Keywords:** Demographic heterogeneity, habitat quality, individual variation, Oyster reef, *Panopeus herbstii*

## Abstract

Variation in habitat quality is common across terrestrial, freshwater, and marine habitats. We investigated how habitat quality influenced the reproductive potential of mud crabs across 30 oyster reefs that were degraded to different extents. We further coupled this field survey with a laboratory experiment designed to mechanistically determine the relationship between resource consumption and reproductive performance. We show a >10-fold difference in average reproductive potential for crabs across reefs of different quality. Calculated consumption rates for crabs in each reef, based on a type II functional response, suggest that differences in reproductive performance may be attributed to resource limitation in poor quality reefs. This conclusion is supported by results of our laboratory experiment where crabs fed a higher quality diet of abundant animal tissue had greater reproductive performance. Our results demonstrate that spatial variation in habitat quality can be a considerable contributor to within-population individual variation in reproductive success (i.e., demographic heterogeneity). This finding has important implications for assessing population extinction risk.

## Introduction

Variation in habitat quality is common across marine, freshwater, and terrestrial systems. This variation can result from numerous forms of human-induced, spatially variable habitat degradation, such as habitat fragmentation resulting from urban development (e.g., Swenson and Franklin 2000), loss or deterioration of wetlands or other natural habitat as a result of human land use changes (Meyer and Turner 1992) or dam construction (Nilsson and Berggren 2000), chemical pollution from both point sources (e.g., Silliman et al. 2012) and nonpoint sources (e.g., Howarth 2008), the accumulation of lost or abandoned fishing gear (UNEP 2005), etc. Alternatively, variation in habitat quality may result from natural spatial variation in geological, physical, chemical, or ecological processes.

Reduced habitat quality commonly has negative consequences for individuals and populations (Sih et al. 2000) due to, among other things, the loss of habitat (Brooks et al. 2002), decreased resource availability, reduced gene flow (e.g., Keller and Largiadèr 2002), or the introduction of toxic substances. Just as the causes of variable, habitat quality may differ, so too can the consequences. At the individual level, degraded habitats can lead to increased mortality risk (e.g., Pettorelli et al. 2003), decreased energetic state (e.g., Craig and Crowder 2005), altered neurological or endocrine function (Homyack 2010), and reduced reproductive performance (e.g., Norris et al. 2004). At the population level, these individual-level effects can result in reduced population growth rates (Sibly and Hone 2002) and increased risk of extinction (e.g., Crooks and Soulé 1999), yielding a loss of biodiversity at the community or ecosystem levels (Fahrig 2003; Wilson et al. 2008).

A primary challenge facing ecology today is to understand how the decline in habitat integrity influences the persistence of populations from a mechanistic point of view. Achieving a mechanistic understanding of the impacts of habitat quality on population persistence is necessary for predicting the consequences of novel or additional future habitat degradation. Similarly, understanding the mechanistic link between habitat quality and population success is equally necessary for predicting the responses of populations to habitat improvements resulting from ecological restoration efforts. Ultimately, changes to population persistence can occur via changes in mortality rates or reproductive success. In this study, we focus on reproductive implications of habitat quality.

The ecological importance of spatial variation in habitat quality depends on its scale relative to the scale of organismal movement. At one extreme, when habitats of differing quality are large and movement between them is limited, this spatial variation in habitat quality can result in source-sink population dynamics (Pulliam 1988). At the other extreme, when the scale of daily organismal movement is broad relative to the scale of spatial variation in habitat quality, organisms often select the highest quality habitats, giving rise under certain conditions to the ideal free distribution of organisms across resource patches (Fretwell and Lucas 1970). Conditions between these two extremes may also occur when the scale of organismal movement is more spatially restricted than the scale of spatial variation in habitat quality, yet individuals across a broad geographic region with habitats of differing qualities are part of a single population. In this case, members of a single population will persistently experience habitats of different qualities, even within a small geographic range. This small-scale variation in habitat quality may be an important driver of between-individual variation in vital rates, with potentially important demographic consequences.

Variation in demographic processes is common. Variation due to temporal changes in environmental processes that influence the entire population is generally captured in population models as environmental stochasticity (Engen et al. 1998). Variation due to differences between individuals is captured in multiple forms. Variation in population growth rates arising from random differences between individual survival and reproduction is captured as demographic stochasticity (Engen et al. 1998), while variation due to differences in vital rates across age, size, or developmental stage is captured as demographic heterogeneity in structured population models (Caswell 2001). However, there are also other important sources of demographic variation that are not as easily measured and so are commonly modeled as stochasticity (described in Kendall et al. 2011). These include genetic variation, maternal effects, or spatial heterogeneity in environmental quality. Despite the difficulty in measuring these factors, they can be major sources of variation leading to extinction risk (Melbourne and Hastings 2008). Finally, stochastic population models often assume that fecundity is Poisson distributed (e.g., Akcakaya 1999). Under these conditions, variation in fecundity among individuals has no effect on demographic variance because when mean = variance, Jensen's inequality is transformed into an equality (Kendall and Fox 2002). However, as previously pointed out (Kendall and Fox 2002), there is no biological justification for using the Poisson distribution to model fecundity, and variance in fecundity that is an accelerating (decelerating) function of the mean would increase (decrease) total demographic variance.

Here, we examine the contribution of spatial heterogeneity in habitat quality in causing individual variation in reproductive effort from a mechanistic perspective. Specifically, we examine how resource abundance differs across a range of habitats in close proximity, but where organismal movement limits individuals to small geographic ranges. We test the hypothesis that a large portion of the between-individual variation in reproductive success is determined by small-scale variation in habitat quality. We also examine the mean–variance relationship in fecundity across reefs of different qualities to test the validity of using the Poisson distribution to model fecundity.

## Methods

### Study system

We examine the mechanistic link between habitat quality and reproductive success using one of the most degraded coastal habitats worldwide. Oyster reefs have declined by 85% from their historic levels (Beck et al. 2009) due to a combination of anthropogenic factors, including harvesting, sedimentation, diseases, introduced pests, and oxygen depletion (Lenihan and Peterson 1998). Remaining oyster reefs are often highly degraded due to combinations of these same stressors. The degradation of oyster reef habitat has cascading impacts that extend beyond the oysters themselves to negatively influence species that utilize these habitats (Lenihan et al. 2001).

**Figure 1:**
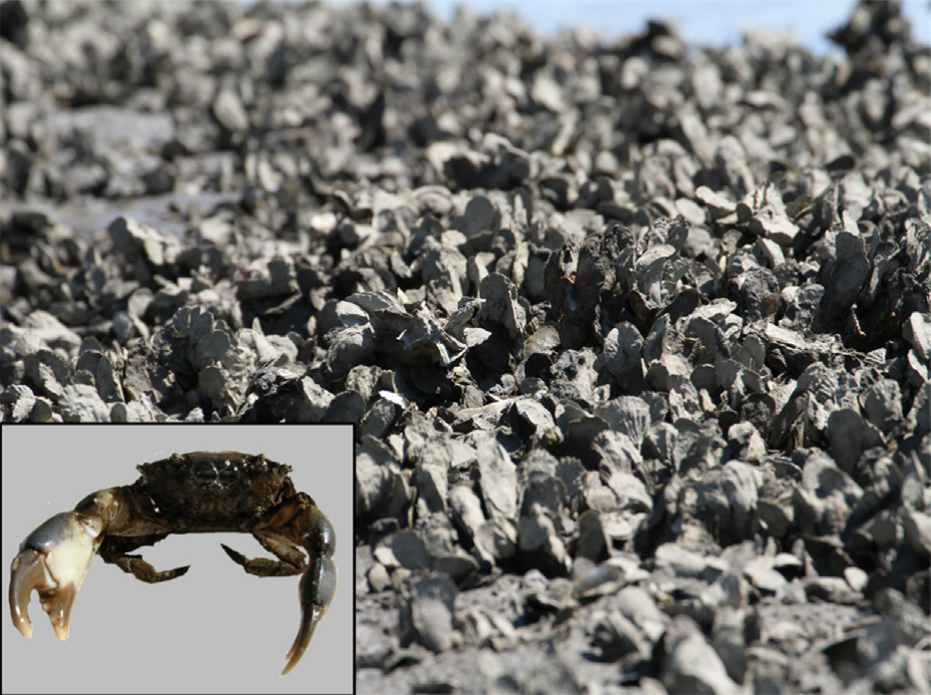
Oyster reef in North Inlet, South Carolina where this study was conducted. *Inset*: Mud crab *Panopeus herbstii*.

The mud crab *Panopeus herbstii* (Fig.1) is a prominent consumer in oyster reef habitats along the Atlantic and Gulf coasts of North America where it primarily consumes small bivalves, including the oyster *Crassostrea virginica* and the scorched mussel *Brachidontes exustus* (Toscano and Griffen 2012). However, the mud crab is omnivorous and consumes numerous food items with different frequencies based on gut content analyses of crabs from this same site: bivalves > barnacles > algae > detritus > crustaceans > marsh grass (Griffen and Mosblack 2011). The mud crab is relatively immobile compared with other species of crab that use the same habitat such as the blue crab *Callinectes sapidus* or the stone crab *Menippe mercenaria*. A previous resampling study found that mud crabs in North Inlet, SC, commonly remain on the same reef for more than a month (Toscano and Griffen 2014). Females reach sexual maturity at approximately 16 mm carapace width (Hines 1989) and generally only produce a single clutch of eggs per year that can be highly variable in size, ranging from 3000 to 113 000 eggs (McDonald 1982). While mud crabs can reproduce from March–October, occurrence of gravid/vitellogenic females peaks in May (McDonald 1982).

We conducted our study in oyster reefs in the North Inlet national Estuarine Research Reserve in South Carolina. This is a saline estuary (34 psu, Dame et al. 1986) with generally healthy oyster reefs that cover extensive areas of intertidal channels.

### Correlation between reef quality and reproductive performance

We sampled 30 reefs varying in complexity and height within North Inlet during May and June 2014. We chose reefs that were close in proximity in order to focus on small-scale variation in habitat quality that was not confounded by environmental changes over larger spatial scales (mean ± SD distance between nearest neighbor reefs 38.7 ± 60.1 m; we determined the distance between all sites using GPS coordinates and the fields package in R (Nychka et al. 2014), and minimum distance between sampling sites were then determined for each reef using the agrmt package in R (Ruedin 2014)). Sampling was conducted during low tide. Reef height is an easily measured metric of reef quality, as the growth and survival of oysters increases with reef height (Lenihan and Peterson 1998). Reefs were chosen based on reef height (range 5–25 cm) in order to ensure relatively even sampling across reefs of different qualities; thus, we do not present the natural distribution of reef heights at our site. Reefs on the lower end of this range represented heavily degraded reefs, while those on the higher end represented thriving, healthy reefs. We determined reef height using the average of 10 measurements taken at random locations within each reef, each one measuring the height from the surface of the mud to the top of the oyster shells. The quality of reefs sampled was randomized through time so that there was no trend between reef height and Julian day in our sampling (*P *=* *0.24).

Within each reef, we conducted sampling within three haphazardly placed 1 m^2^ quadrats. From each quadrat, we collected all seaweed (primarily *Ulva* spp.) and all mature-sized female mud crabs (i.e., those >16 mm carapace width). Algae and crabs collected from each reef were returned to the Baruch Institute for Marine and Coastal Sciences laboratory where algae was rinsed, dried for 72 h at 70°C, and weighed. Additionally, we counted the number of small bivavles (*C. virginica* and *B. exustus*) within two 0.5 m^2^ quadrats on each reef. We defined small bivalves to be those <4 cm in shell length. We explored spatial autocorrelation in the density of small bivalves across reefs using Moran's I (Diniz-Filho et al. 2003) from the ape package in R (Paradis et al. 2004). This test provides a statistic that ranges from 0 (completely random) to 1 (perfectly spatially autocorrelated). We used a generalized linear model with a Poisson distribution to determine which environmental factors influenced the number of gravid or vitellogenic crabs collected in quadrats at each reef. We combined gravid and vitellogenic crabs as the response variable in this analysis because this reflects all crabs engaged in reproduction at the time of sampling. As predictor variables, we used reef height, the density of small bivalves, algal biomass, Julian day, longitude, and latitude. We also used a linear model to determine how log bivalve density varied with reef height.

Collected crabs were sacrificed by placing them into a freezer overnight, after which they were measured (carapace width) and dissected. We removed extruded egg clutches, ovaries, and the hepatopancreas. These, along with the remainder of the body, were each dried separately for 72 h at 70°C and weighed to the nearest 0.01 mg. Some of the crabs sampled were gravid, while others were in the process of producing eggs (vitellogenic), but had not yet completed this process. Energy dedicated to reproduction in crabs is generally withdrawn from the energy stored in the hepatopancreas (Anilkumar 1980). We therefore analyzed extruded eggs, ovaries, and the hepatopancreas together using the gonado-hepatosomatic index (GHSI), determined by dividing the combined mass of eggs, ovaries, and hepatopancreas by the mass of the rest of the crab. We used a linear model to determine how the GHSI (averaged for all crabs on a single reef) varied across reefs as a function of the density of small bivalves (log transformed), Julian day, and algal abundance. We used the number of crabs collected from each reef as a weighting factor in the analysis to account for different sample sizes across reefs.

We used a predator-dependent functional response equation to calculate the consumption rate (*C*) for crabs within each of the 30 reefs as a function of small bivalve density (*N*) and crab density (*P*). This calculation assumes that oyster and mussel prey can be combined to a single value of *N* to determine a single functional response for bivalves in general. It also does not account for differences in structural complexity of oyster reefs that are known to influence the functional response of mud crabs (Toscano and Griffen 2013). Nevertheless, it provides a rough approximation of likely relative consumption rates of crabs across our sampled reefs that had widely different numbers of bivalve prey. The type II functional response was as follows:

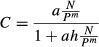


where *a* is the search efficiency (set to 1.534 based on the fit of this model to empirical data of mud crabs foraging on scorched mussels, Toscano and Griffen 2013), *h* is the handling time (set to 0.1414 days, Toscano and Griffen 2013), and *m* is the interference coefficient and can range from 0 (prey dependent) to 1 (ratio dependent) or >1 (predator density is more important than prey density in determining consumption rates). The value of this coefficient is species specific (Griffen and Delaney 2007), and is unknown for *P. herstii* and depends on the strength of conspecific predator interference. Two pieces of evidence suggest that the value of this coefficient should be intermediate for *P. herstii*. First, mud crabs do aggregate at high densities in oyster reefs, suggesting that interference is not too strong. Second, mud crabs will readily attack each other and there is a high incidence of nonlethal injury (for instance, 39.5% of our sampled crabs were missing limbs). Thus, we chose a middle of the road value of *m *=* *0.5 (we also examined the sensitivity of our results to the value of *m* by repeating calculations using *m *=* *0.25 and *m *=* *0.75 to see how this would change our results). Finally, we used a linear model to demonstrate how the calculated consumption rate (*C*) changed with the log of the small bivalve density across our 30 sampled reefs.

An assumption inherent in using a Poisson distribution to model fecundity is that mean = variance. We used the sampling data to test this hypothesis. We used GHSI values measured on each crab here to get a rough approximation of the number of eggs produced by each crab. Not all energy stored in the hepatopancreas will ultimately support reproduction, some will undoubtedly instead contribute to individual growth (i.e., molting, O'Connor and Gilbert 1968), while an additional small amount is likely reserved in both the ovaries and the hepatopancreas following reproduction. However, for simplicity, if we assume that the entire contents of the gonad+hepatopancreas is allocated to egg production, then we can calculate a relative fecundity for each crab. We made this calculation by dividing the mass of the gonad+hepatopancreas by the mass of a single egg (5.5 *μ*g, McDonald 1982). For each reef, we next calculated the mean and the variance of fecundity for all crabs collected from that reef. We then determined the relationship between variance (*y*) and mean (*x*) fecundity across reefs using two statistical models. The first was a simple linear model (*y* = *ax + b*), a test that mean = variance when *a *=* *1 and *b *=* *0. The second was a power model (*y* = *ax*^*b*^) that would allow detection of a concave relationship when *b *<* *1.0, while *b *>* *1.0 would indicate a convex relationship. Finally, we compared the fit of these two models using AIC.

Lastly, we used the fecundity for each crab calculated above to approximate the relative total number of eggs produced across reefs of different quality. We did this by summing individual potential egg production across all the crabs on a single reef.

### Experimental test of link between diet and reproduction

In order to understand the mechanistic, causative link between consumption and reproductive performance in the absence of other energetic constraints or costs (e.g., search or handling energetic costs, predation risk, etc.), we conducted a laboratory study in which we quantified consumption and reproductive effort. We collected 40 female mud crabs in May (20.9–34.05 mm carapace width) from North Inlet. These were returned to the University of South Carolina in Columbia where they were placed into a recirculating aquarium held at 20°C on a 16 h:8 h light:dark cycle. Each crab was housed in an individual 1-L chamber, individually plumed to ensure constant flow at 3 L/h. Crabs were fed twice per week (Monday and Thursday) and were given 48 h to consume their food before uneaten food was removed, dried for 72 h at 70°C, and weighed to the nearest 0.01 mg. Crabs were fed one of 20 experimental diets that varied the total amount of food present (0.3, 0.6, 1.2, 3% of body weight at each feeding) and the proportion of that food that was animal tissue or algae (0.0:1.0, 0.25:0.75, 0.5:0.5, 0.75:0.25, 1.0:0.0 animal:algal). Two crabs were fed each of these 20 experimental diets. However, food consumed could not be directly controlled (food offered served as an upper limit to consumption, but individual crabs could always choose to consume less on any given day). Each crab therefore had a different diet over the course of the experiment that reflected its aggregate daily diet decisions. We determined the average animal and algal consumption across feeding periods for each crab over the course of the experiment and use these average values as a continuous predictor variable in statistical analyses described below. In preliminary experiments, we found that mussels and oyster tissues disintegrated and resulted in high nonconsumptive food losses over a single feeding period. We therefore chose to use tilapia as an alternative food source because it does not disintegrate as readily, and has similar energetic and nutrient content to mussels (Griffen 2014). We used *Ulva lactuca* as an algal food source because it is common in tidal channels and is often found snagged on shells in oyster reefs (Griffen, pers. obs.) and because *Ulva* is the preferred algal food of *P. herbstii* (Stachowicz and Hay 1999).

This experiment continued as described above for 10 weeks, after which the crabs were dissected and ovaries, hepatopancreas, and the rest of the body were dried separately at 70°C for 72 h and were then weighed to the nearest 0.01 mg. To be consistent with the field sampling data described above, we determined the relative reproductive effort of experimental crabs using the GHSI. We used a linear model to determine how the GHSI varied with the mass-specific average daily consumption of animal tissue and with the mass-specific average daily consumption of algae. A plot of GHSI versus animal consumption suggested a saturating relationship. We therefore also fit a second-order polynomial to the data and compared these two models with AIC. Three crabs molted during this experiment. We therefore also included molting as a binary predictor variable (1/0) in these analyses.

## Results

### Correlation between reef quality and reproductive performance

**Figure 2:**
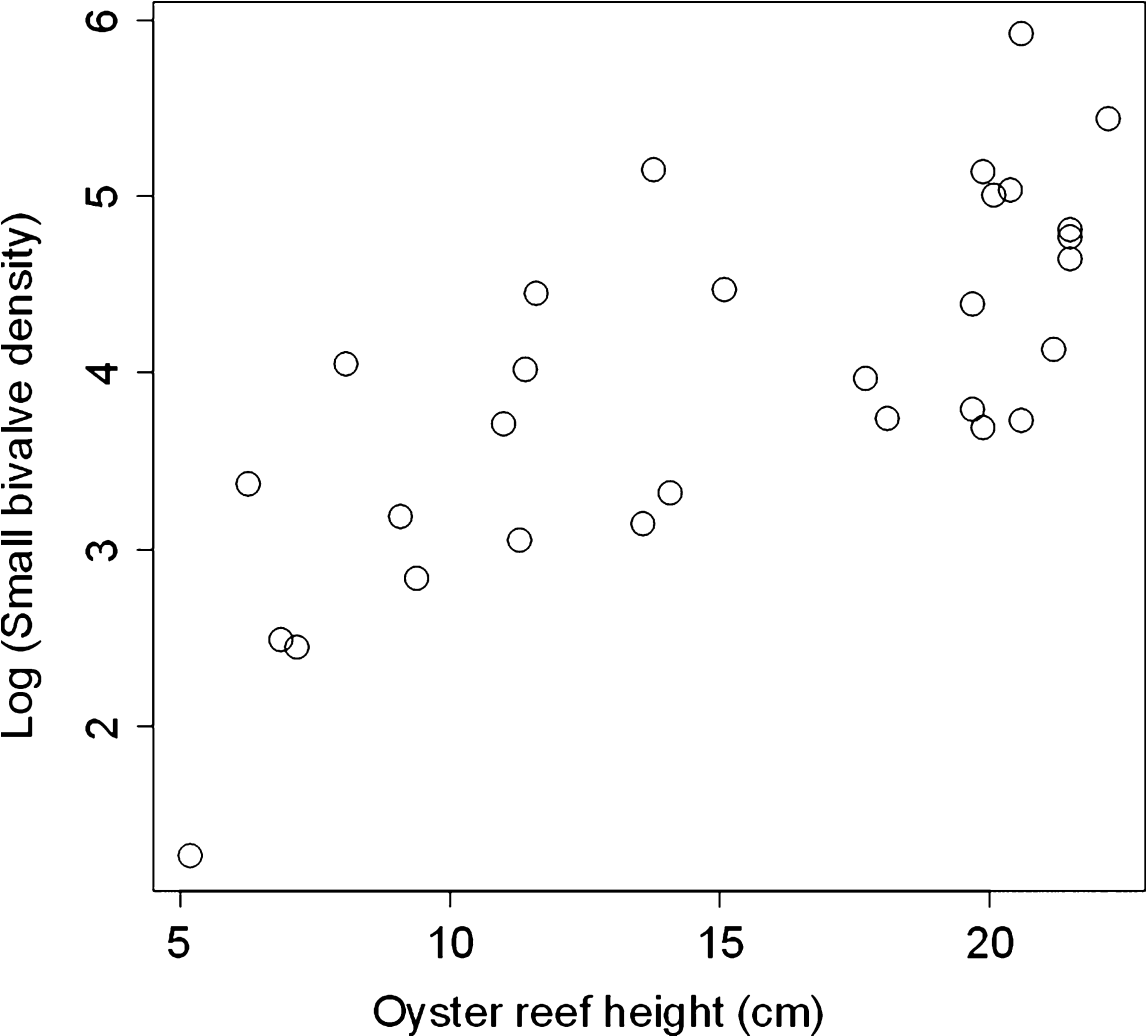
The density of small bivalves (oysters and mussels <4 cm shell length) increased with oyster reef height.

**Figure 3:**
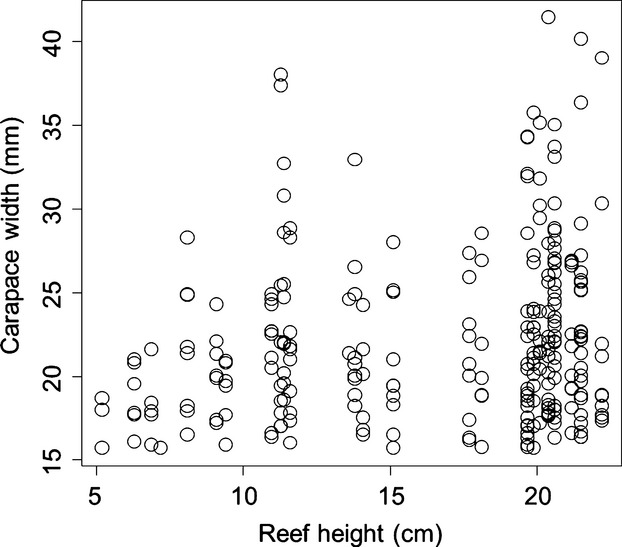
Distribution of carapace widths for large crabs (>16 mm CW) across reefs of different height, demonstrating that larger crabs generally resided in taller reefs.

**Figure 4:**
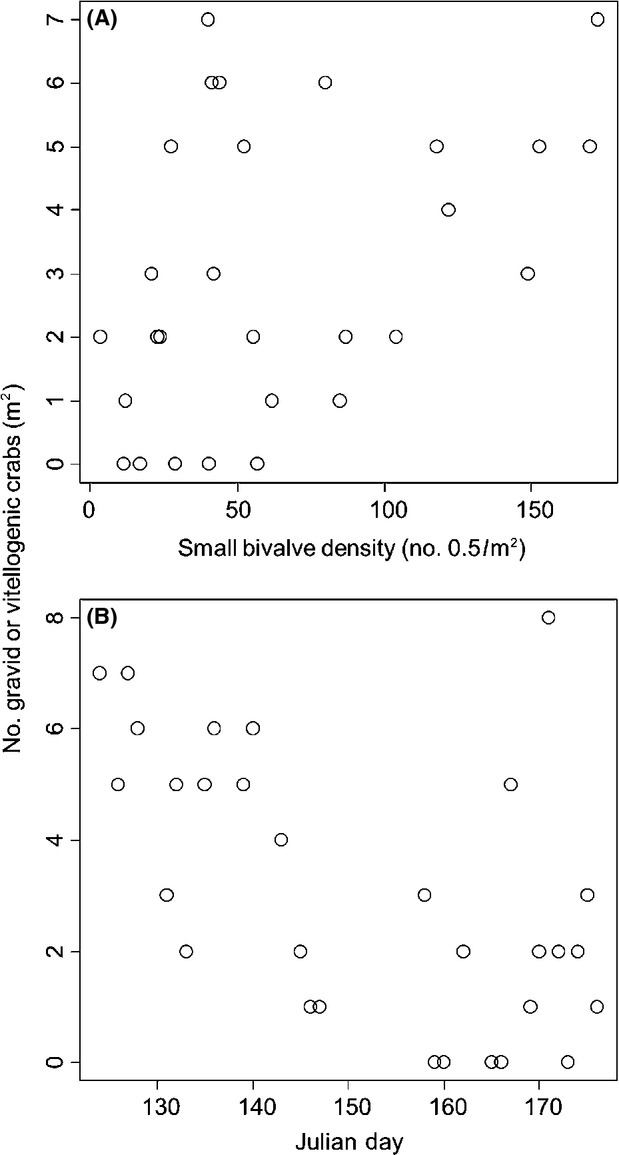
The number of reproductive (gravid+vitellogenic) crabs increased with the density of small bivalves (part A) and decreased with time throughout our sampling period (part B).

The density of small bivalves (scorched mussels+oysters) ranged from 4 to 370 bivalves per 0.5 m^2^ (mean ± SD = 81.4 ± 79.0), but was not autocorrelated across sites (Moran's *I* = 0.097, *P *=* *0.092). We found across reefs that the log abundance of small bivalves increased by 0.133 ± 0.023 each 1-cm increase in reef height (*t* = 5.73, *P *≪ 0.0001, adjusted *R*^2^ = 0.52, Fig.2). Across all 30 reefs, we sampled a total of 264 reproductive-sized female crabs. There was a general increase with reef height in the number of mature-sized crabs and the maximum size of crabs observed (Fig.3). Of the 264 crabs sampled, 51 were gravid and 43 were vitellogenic. Overall, we found that the number of reproductive crabs (i.e., gravid+vitellogenic) on a reef increased by 0.085 ± 0.032 with each 1-cm increase in reef height (*z* = 2.66, *P *=* *0.008) and increased by 0.004 ± 0.001 for each additional small bivalve present (*z* = 2.71, *P *=* *0.007, Fig.4A), decreased by 0.032 ± 0.011 with each passing day during the sampling period (*z* = −3.04, *P *=* *0.002, Fig.4B), but was not influenced by algal abundance (*z* = −1.57, *P *=* *0.118), or by location (longitude: *z* = 0.14, *P *=* *0.89; latitude: *z* = −0.78, *P *=* *0.43).

**Figure 5:**
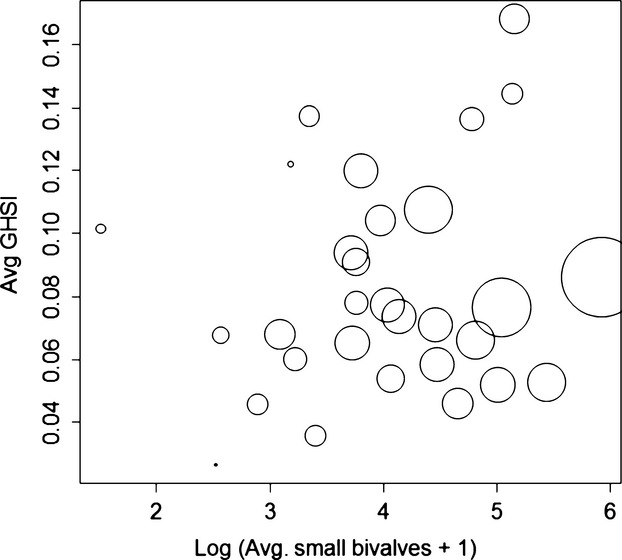
Average reproductive potential (gonado-hepatosomatic index, GHSI) of all large crabs (>16 mm CW) in a reef increased with the density of small bivalves in that reef. Circle size indicates the relative number of crabs captured in each reef.

**Figure 6:**
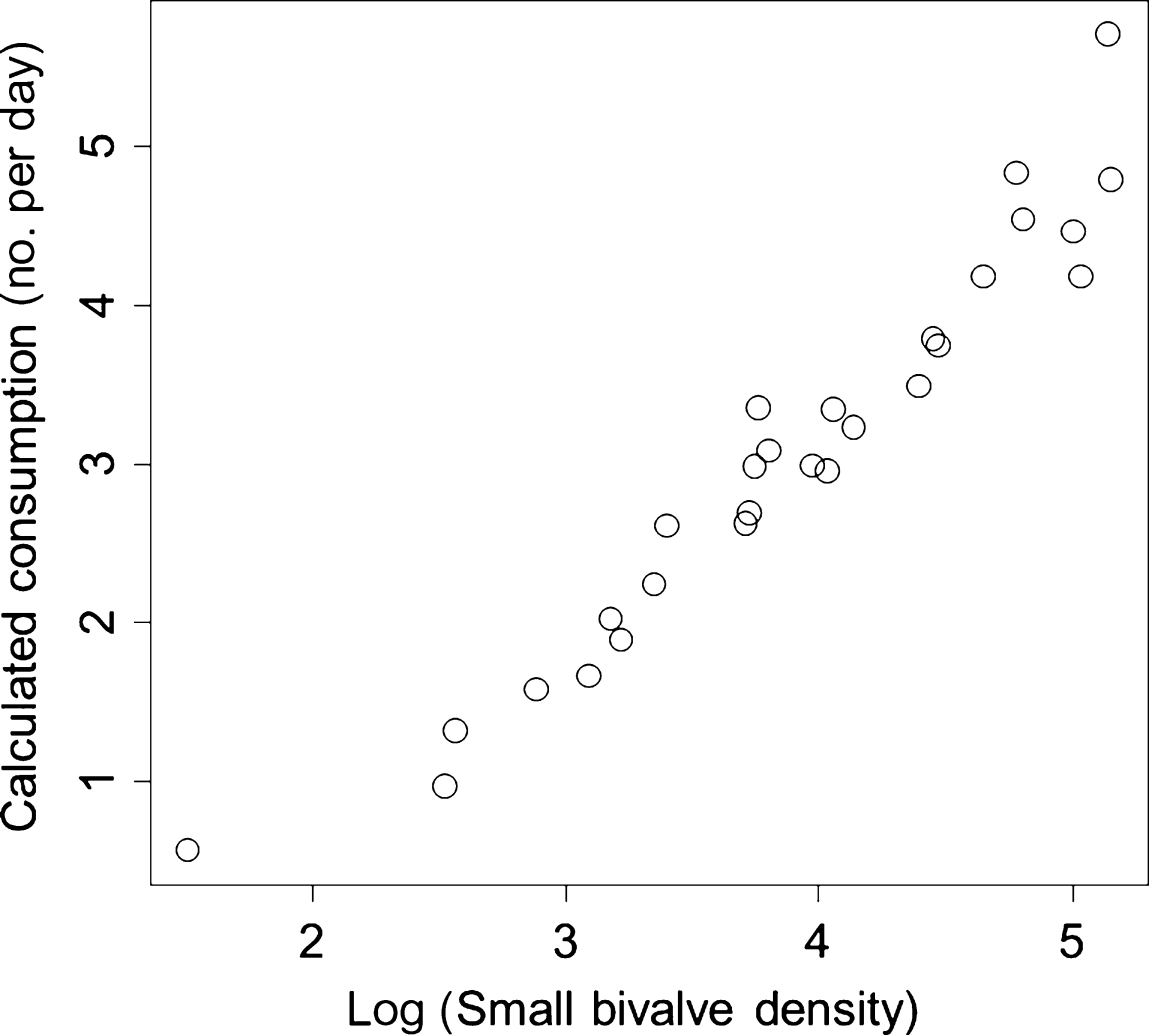
Increasing consumption rate as a function of small bivalve density calculated using a type II functional response and published empirically determined search efficiencies and handling times for this system.

**Figure 7:**
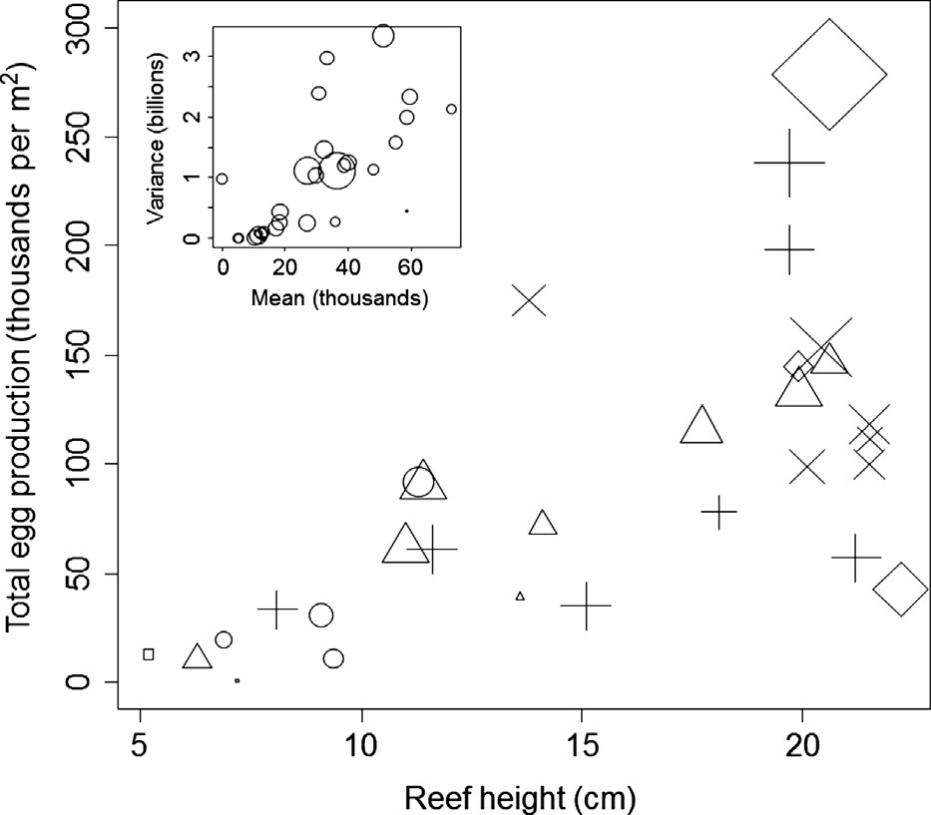
Total calculated egg production by all mature crabs across 30 sampled reefs as a function of reef height. Symbols represent calculated consumption rates given in Figure6, rounded down to the nearest whole bivalve consumed per day (square = 0, circle = 1, triangle = 2, cross = 3, × = 4, and diamond = 5 per day). Symbol size represents the relative number of large crabs captured on each reef. *Inset*: Relationship between the mean number of eggs per crab and the variance in the number of eggs per crab at each reef. Circle size is relative number of large crabs captured on each reef (symbol sizes on main figure and in inset figure are at different scales).

We found that the average GHSI (a size-independent measure) increased by 0.012 ± 0.004 across reefs with the log abundance of small bivalves (*t* = 2.95, *P *=* *0.007, adjusted *R*^2^ for overall analysis = 0.62, Fig.5), decreased by 0.001 ± 0.0002 with Julian day (*t* = −6.26, *P *≪ 0.0001), but was not influenced by the abundance of algae (*t* = −0.24, *P *=* *0.809). Using partial linear regression, we found that the density of small bivalves accounted for 19.7% of the overall variation in GHSI across reefs. Using a type II functional response, we calculated that the likely consumption rate of crabs should increase by 1.334 ± 0062 bivalves per day with the log of the bivalve density across reefs (*t* = 21.62, *P *≪ 0.0001, Fig.6). This estimate changed by <15% when *m* varied between 0.25 and 0.75. Finally, the total calculated egg production on each sampled reef increased as a function of reef height (Fig.7), and the variance in egg production by each crab increased faster than the mean (linear model AIC = 1268.76, power model AIC = −378.56, *b *=* *1.117, *P *=* *0.0003, Fig.7 inset). Overall variance in potential egg production across all sampled crabs (1 255 936 937) was more than 40 000× greater than the overall mean potential egg production (31 340 eggs).

### Experimental test of link between diet and reproduction

**Figure 8:**
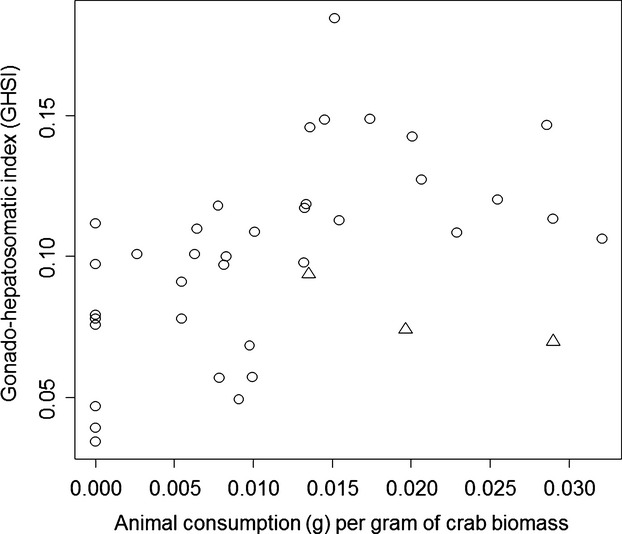
Asymptotically increasing reproductive potential (GHSI) with the average daily amount of animal tissue consumed per gram of crab in a 10-week laboratory feeding experiment. Triangles represent three crabs that molted during the experiment.

In our laboratory experiment, algal consumption increased by 0.11 ± 0.02 g for each additional 1.0 g offered (*t* = 7.06, *P *≪ 0.0001, adjusted *R*^2^ = 0.57). Similarly, animal tissue consumption increased initially by 0.12 ± 0.007 g for each additional 1.0 g offered (*t* = 14.9, *P *≪ 0.0001, adjusted *R*^2^ = 0.93); however, animal consumption saturated at high amounts offered (second-order polynomial term: *t* = −7.88, *P *≪ 0.0001, AIC of linear first-order model −241.26, AIC of second-order model −278.04). Twenty-three of the 40 experimental crabs were vitellogenic at the conclusion of the experiment. Reproductive effort and energy storage, as indicated by the GHSI, increased in a saturating manner with animal tissue consumed (first-order polynomial model term *t* = 3.43, *P *=* *0.002; second-order polynomial model term *t* = −2.54, *P *=* *0.016; AIC of first-order model −160.98, AIC of second-order polynomial −165.75, Fig.8), and decreased for the three individuals that molted (*t* = −2.36, *P *=* *0.024, Fig.8), but was not influenced by algal consumption (*t* = 0.98, *P *=* *0.332).

## Discussion

We have demonstrated that reproductive effort varies over relatively small spatial scale (on the order of meters) across habitats with different amounts of food resource. Results of our laboratory experiment support the conclusion that these differences in reproductive effort were likely a direct result of diet limitation in reefs with few resources. These results have important implications both for this study system and for ecology more broadly.

### Implications for this study system

Our results suggest large differences in the contribution of different individuals to population success that are driven largely by differences in habitat quality. These results are consistent with a previous report that found egg number in gravid females to vary widely, from 3000 to 113 000 between crabs (McDonald 1982). Overall, we found that ∽20% of the variation in average GHSI across reefs was explained by differences in food availability. Additional unexplained variation may have been caused by movement of crabs between reefs of different quality prior to sampling. While mud crabs can remain on a single reef for many weeks at a time (Toscano and Griffen 2014), another previous study found that they may move >5 m over 48 h (Stachowicz and Hay 1999). Average distance between our sampled reefs was ∽39 m.

We calculated an overall increase in egg production in higher quality reefs (we used reef height as a proxy for reef quality as this is the easiest thing to measure in the field). This increase in egg production was due to a combination of three factors. First, higher quality reefs had a greater number of reproductive-sized crabs overall. Second, although all crabs examined here were of reproductive size, taller reefs generally had larger crabs, and egg production increases with crab size (Hines 1982). And third, for any given size crab, higher quality reefs generally supported higher energy stores (GHSI), likely due to the greater abundance of small bivalve prey that facilitated increased consumption rates. The fact that most egg production comes from healthy, high-quality reefs implies that the success of the mud crab population is dependent on the persistence of high-quality reefs and that mud crab populations should not be expected to thrive if low-quality reefs are the norm, even if those low-quality reefs are extensive.

Predation mortality for mud crabs is higher in simple reefs than in more complex reefs (Grabowski 2004). Thus, spatial differences in habitat quality will also result in spatial variation in mortality rates. The relative importance of spatial variation in reproduction and in mortality is unclear.

### Broader implications

Results presented here have broader implications for stochastic population modeling. As described in the Introduction, an incomplete understanding of processes that cause demographic heterogeneity has commonly resulted in the use of the Poisson distribution to stochastically model this variation. For the process that creates this heterogeneity, this approach implicitly assumes that the mean is equal to the variance (i.e., that mean fecundity across individuals is equal to the variance in fecundity). We have shown for our study system that this assumption does not hold. Specifically, we found that variance ≫ mean for fecundity and that the relationship between the two was convex. This means that between-individual variation in fecundity in this or other similar systems would increase demographic variance that is an important contributor to population extinction risk (Kendall and Fox 2002; Melbourne and Hastings 2008).

Our study examined reproductive variation resulting from spatial variation in habitat quality. Spatial variation in habitat quality is a common phenomenon in a diverse range of natural systems. For instance, it is common in marine and forest systems where recruitment of larvae (i.e., food for other species) and seed dispersal are highly spatially variable (Gaines et al. 1985; Wright et al. 2005). Spatially variable human impacts may also make environments more heterogeneous over small scales leading to spatial variation in demographic factors as we have documented here. Similarly, spatial variation in habitat quality in other systems can strongly influence both reproductive success (Wiehn and Korpimäki 1997) and mortality risk (Stevens and Baguette 2008). In contrast to demographic stochasticity whose influence is inversely correlated with population size (Morris and Doak 2002), demographic variation driven by differences in habitat quality may become more important as population sizes increase and high-quality habitats become saturated, forcing individuals to take up residence in habitats of lower quality. In this case, this variation reflects structured individual variation (sensu Kendall and Fox 2003), or vital rates in one individual that are not independent of vital rates in other individuals.

Habitat degradation is an important driver of population extinction risk (Griffen and Drake 2008; Drake and Griffen 2010), and understanding and accurately modeling the contribution of demographic heterogeneity to total demographic variation is crucial for accurately assessing this extinction risk (Melbourne and Hastings 2008). The strength of environmentally driven demographic heterogeneity (i.e., the difference between the “haves” and the “have not's”) should increase with the severity of habitat destruction or modification. This has implications for how this variation should be modeled. As described above, an incomplete knowledge of demographic heterogeneity often results in lumping this variation as a component of demographic stochasticity, and modeling it as variation around the mean that is nondirectional using a Poisson distribution. We have shown that for systems like ours, this is an inappropriate error structure because variation in fecundity is much greater than mean fecundity. But this modeling approach may also be inappropriate for a second reason. Environmental degradation is a directional shift in quality through time, and when this degradation is spatially variable, it should always lead to an increase through time in variation in vital rates across habitat patches. This means that variation in vital rates around mean values will be directional rather than truly stochastic. When environmental factors are a dominant source of demographic variation, it may therefore be preferable to model this variation using a more appropriate modeling structure that accounts for changes in the amount demographic heterogeneity over time as habitats become more degraded.

Finally, we note that demographic heterogeneity driven by spatial differences in habitat quality may interact with large-scale temporal changes in environmental quality that are modeled as environmental stochasticity. Environmental stochasticity derives from factors that influence the entire population simultaneously, producing good years and bad years, for example. However, good and bad years may not influence all individuals in a population equally when individuals already experience very different environmental conditions due to spatial variation in habitat quality. Thus, a good year may have a large positive influence on individuals in poor quality habitats, while its effects may be minimal on individuals in high-quality habitats if there is an upper limit to resource utilization (e.g., an asymptotic consumption rate as is commonly seen in a type II or type III functional response). Similarly, a bad year may have much larger impacts on an individual whose poor habitat quality has left them in poor physiological condition than on individuals with large energetic reserves resulting from their occupancy of high-quality habitats.
